# Differential Frequency of CD8+ T Cell Subsets in Multiple Sclerosis Patients with Various Clinical Patterns

**DOI:** 10.1371/journal.pone.0159565

**Published:** 2016-07-28

**Authors:** Zahra Salehi, Rozita Doosti, Masoumeh Beheshti, Ehsan Janzamin, Mohammad Ali Sahraian, Maryam Izad

**Affiliations:** 1 Immunology Department, School of Medicine, Tehran University of Medical Sciences, Tehran, Iran; 2 MS Research Center, Neuroscience Institute, Tehran University of Medical Sciences, Tehran, Iran; 3 Pathophysiology laboratory, Sina hospital, Tehran University of Medical Sciences, Tehran, Iran; 4 Flow Cytometry laboratory, Department of Stem Cell and Developmental Biology, Royan Institute, Tehran, Iran; Charite Universitätsmedizin Berlin, GERMANY

## Abstract

Recent evidence points to a pathogenic role for CD8^+^ cytotoxic T (Tc) cells in Multiple sclerosis (MS). Based on cytokine profile, Tc cells can be divided into different subsets: IFN-γ (Tc1), IL-4 (Tc2), IL-10 (Tc10), IL-17 (Tc17), IL-21 (Tc21), IL-22 (Tc22) and TNF-α producing cells. In this study we evaluated the frequency of Tc cell subsets and the serum level of Tc17 differentiation cytokines in MS patients with different clinical patterns. We analyzed Tc cell subsets percentage in peripheral blood of relapsing-remitting (RRMS) (n = 28), secondary-progressive (SPMS) (n = 10) and primary-progressive (PPMS) (n = 4) MS patients in comparison to healthy controls (n = 15) using flow cytometry. Serum level of TGF-β, IL-6 and IL-23 were measured by ELISA. We showed elevated levels of Tc1 and Tc17 cells in SPMS and RRMS patients in relapse phase, respectively (P = 0.04). Interestingly, the percentage of TNF-α producing CD8^+^ T cells in relapse and remission phase of RRMS and SPMS patients were higher than controls (P = 0.01, P = 0.004, P = 0.01, respectively) and Tc21 increased in remission phase of RRMS compared to SPMS (P = 0.03). We also found higher frequency of CD8^+^ IFN-γ^+^ TNF-α^+^ IL-17^+^ T cells in relapse phase of RRMS compared to remission phase, SPMS patients and controls (P = 0.01, P = 0.004 and P = 0.02, respectively). TGF- β increased in sera of RRMS patients in remission phase (P = 0.03) and SPMS (P = 0.05) compared to healthy subjects. Increased level of Tc17 and CD8^+^ IFN-γ^+^ TNF-α^+^ IL-17^+^ T cells in relapse phase highlights the critical role of IL-17 in RRMS pathogenesis.

## Introduction

Multiple sclerosis (MS) is a chronic demyelinating disease of the central nervous system (CNS) [1] which is affecting more than 2.5 million people worldwide, mostly young people [2]. According to Etemadifar et al. study, Iran has a medium-to-high prevalence rate of MS and it seems to have significantly increased during recent years [3]. Almost 85% of patients show a relapsing-remitting multiple sclerosis disease pattern (RRMS). However, a secondary progressive multiple sclerosis (SPMS) strikes in most RRMS cases after ten years. Approximately 10–15% of patients show a primary progressive multiple sclerosis (PPMS) which is characterized by the disease progression from the onset [4].

It is believed that autoimmune response to self-antigens plays a major role in MS pathogenesis in a genetically susceptible individual [5]. Due to increased frequency of Th1 and Th17 cells in periphery and inflamed CNS of both MS patients in relapse phase and Experimental Autoimmune Encephalomyelitis (EAE), it has been proposed for a long time that Th1 and Th17 cells are the hallmarks of MS pathogenesis [6–8]. However, recently many studies have speculated that CD8^+^ cytotoxic T cells have a critical role in MS [9, 10]. Investigation on the brain tissues of 16 progressive and 2 acute MS patients has shown that active demyelinating plaques contained up to 50 times more CD8^+^ than CD4^+^ T cells [11]. Compared to healthy controls, elevated level of CD8+T cell responses to myelin basic protein (MBP) with increased cytotoxicity function has been observed in RRMS and SPMS patients. These cells produced TNF-α and IFN-γ in response to MBP [12]. Moreover, inhibition of CD8+ T cells besides CD4+ T cells is beneficial to decrease the relapse rates in MS patients [13].

It is well established that like CD4+ T cells, CD8+ T cells can also be divided into different subsets according to their cytokine profile, IFN-γ (Tc1), IL-4 (Tc2), IL-10 (Tc10), IL-17 (Tc17), IL-21 (Tc21), IL-22 (Tc22) and TNF-α producing cells. There are few studies looking at the frequency of CD8^+^ T cells subsets based on their cytokine profiles. These studies mainly focused on Tc1, Tc2 and TNF-α producing CD8^+^ T cells in MS patients [14–18]. However, increased frequencies of both CD4^+^ IL-17^+^ and CD8^+^ IL-17^+^ T cells have been shown in acute MS lesions [19] and peripheral blood [20] of RRMS patients.

IL-6, TGF-β and IL-23 are among essential cytokines for development and differentiation of Tc17 cells [21]. Ciric et al showed that TGF-β1 plus IL-6 induced IL-17A secretion from Tc17 cells while IL-23 induced pathogenic Tc17 [22]. Studies regarding serum levels of IL-6, IL-23 and TGF-β in MS patients have shown inconsistent results [23, 24]. Taken together, it appears that different CD8^+^ T cell subsets including Tc1 and Tc17 are involved in promoting pro-inflammatory responses in MS. Thus, we have examined the frequency of Tc cell subsets in peripheral blood of MS patients with different clinical patterns. Furthermore, in subsequent experiment we assessed TGF-β, IL-6 and IL-23 serum level and their correlation with Tc17 cells.

## Material and Methods

### Patients and Controls

Forty-two MS patients (37 females, 5 males; mean age: 33.67±8.48) with clinically definite MS, according to the revised McDonald's criteria (McDonald et al., 2010)[25] (28 RRMS, 4 PPMS and 10 SPMS) were enrolled in this study. Fourteen RRMS patients were in relapse phase with predefined inclusion criteria of not being treated with any kind of interferon beta (IFN-β) and corticosteroid for at least 3 prior months. A relapse was defined as occurrence of new symptoms which lasted at least 24h. Fourteen RRMS patients were in remission phase and had to be relapse free. RRMS patients in remission phase were either using no immune modulatory drugs or IFN-β therapy. PPMS and SPMS patients had active progression. Exacerbation and progression were approved by experienced neurologist. For each patient, the type of MS was determined according to the Lublin–Rheingold classification. Disability was assessed by an experienced neurologist using expanded disability status scale (EDSS). Fifteen ethnically matched individuals (10 females, 5males; aged: 28.6±8.08), who had no history of MS or other inflammatory diseases in their families, were signed up as healthy controls. The inclusion and exclusion criteria of participation in the study are listed in Table 1. All patients and controls were Iranian Caucasian origin. The study was approved by ethics committee of Tehran University of Medical Sciences (TUMS) and written informed consent was obtained from each study subject prior to the study. All of the patients were referred to Iranian Center of Neurological Research in Sina General Hospital, Tehran University of Medical Sciences, Tehran, Iran.

**Table 1:** Inclusion and exclusion criteria of study subjects.

*Subjects*	*Inclusion criteria*	*Exclusion criteria*
**Patients**	RRMS in Relapse phase (New cases)	History of immunosuppressive/ immune modulatory drugs in 3 months prior to sampling
	RRMS in Remission phase	Other types of MS
	PPMS	--------
	SPMS	--------
**Controls**	Healthy males and females	History of MS or other inflammatory diseases/allergy or cold at the time of sampling

### Cell preparation and culture

Peripheral blood mononuclear cells (PBMCs) were isolated from whole blood by Ficoll density gradient centrifugation using Lymphodex (innotrain, Germany). To assess the intracellular CD8^+^ T cell cytokine profile, PBMCs were cultured at a concentration of 10^6^ cells/well in RPMI1640 supplemented with 10% fetal calf serum (FCS), 1% L-glutamine and 100 U/mL penicillin, and 100 mg/mL streptomycin. Cells were stimulated with functional grade purified anti-human CD3 (200ng/ml) and anti-human CD28 (400ng/ml) (eBioscience, US). Incubation was performed for 66 hours in 5% CO2 at 37°C. Cells were additionally incubated in the presence of 500 ng/mL calcium ionomycine (Sigma Aldrich, US), 50 ng/mL Phorbol 12-myristate 13-acetate (PMA) (Sigma Aldrich, US) and 10 ug/mL of brefeldin A (BD GolgiPlug^™^, US) for 6 h. After incubation time cells were harvested for flow cytometric analysis.

### Multicolor Flow Cytometry Assay

To analyze the frequency of CD8+ T cell subsets, the harvested cells were washed once with PBS and evaluated by flow cytometry. In brief, the 10^6^ cells/100ul staining buffer (PBS + 2%FCS) were first stained with Amcyan-conjugated anti-human CD8 monoclonal antibodies (BD Biosciences, US) for 30 minutes in the dark at 4°c. After surface staining, the cells were fixed and permeabilized using LEUCOPERM^™^ (BIO-RAD, BUF09B, US) according to the manufacturer’s instructions. The cells were stained with anti-IFN-γ-FITC, anti-TNF-α-PE-Cy^™^7, anti-IL-10-eFluor^®^450, anti-IL-17-PE, anti-IL-21-eFluor^®^660, anti-IL-22-PerCP-eFluor^®^710 (eBioscience, US) and anti- IL-4-APC-Cy^™^7 (BioLegend, US).

### Enzyme-Linked Immunosorbent Assay (ELISA)

For investigation of cytokines related to Tc17 development, sera of patients and controls were collected from clot tubes by centrifugation at 1500rpm for 10 minutes at 4°C. Serum samples were stored at –70°C untile the day of test. The concentrations of IL-6 (BD Biosciences, US), IL-23 (e-Bioscience, US) and TGF-β (USCN Life Sciences Inc. HK) were assessed by ELISA following the manufacturer’s instructions. The lower limit of detection for each cytokine was: 9 pg/ml for IL-6; 15 pg/ml for IL- 23; 26.4 pg/ml for TGF-β, as determined by the manufacturer.

### Statistical Analyses

The Statistical Package for Social Sciences (SPSS) statistical software version 21.0 (SPSS Inc; Chicago, IL, USA) was used for data analyses. Data are presented as mean ± SD. A p-value ≤ 0.05 was considered statistically significant. All numeric variables were tested for normality of distribution by the Kolmogorov–Smirnov test. A one-way analysis of variance (ANOVA) method was used to compare the differential frequency of CD8^+^ T cell subsets between groups. If significant differences were found, then the data was either analyzed by Tukey or Bonferroni tests for pairwise comparisons. Associations between the IL-6, IL-23 and TGF-β serum levels and the Tc17 cells frequency were assessed with the Pearson correlation.

## Results

Forty-two MS and 15 healthy subjects were examined in this study. Demographic characteristics of these subjects are given in Table 2.

**Table 2:** Demographic characteristics of patients and controls.

*Characteristics*	*RRMS in Relapse phase*	*RRMS in Remission phase*	*SPMS*	*PPMS*	*HCs*
**Number of subjects**	14	14	10	4	15
**Sex; Female/male**	14/0	13/1	8/2	2/2	10/5
**Age; years**	31.31 ± 8.27	35.07 ± 7.07	35.50 ± 6.62	35.67 ± 6.37	28.6 ± 8.08
**Disease duration; years**	5.37 ± 6.13	6.76 ± 4.38	8.88 ± 4.22	8.66 ± 8.14	_
**EDSS score**	3.29 ± 0.85	2.15 ± 0.55	5.11 ± 1.46	5.00 ± 1.32	_
**Disease severity**	1.46 ± 1.11	0.81 ± 1.29	0.83 ± 0.65	1 ± 0.88	_

Data are expressed as mean ± SD.

HC = Healthy Controls; MS = Multiple Sclerosis; EDSS = Expanded Disability Status Scale

Disease severity: EDSS score/Disease duration

**Fig 1:**
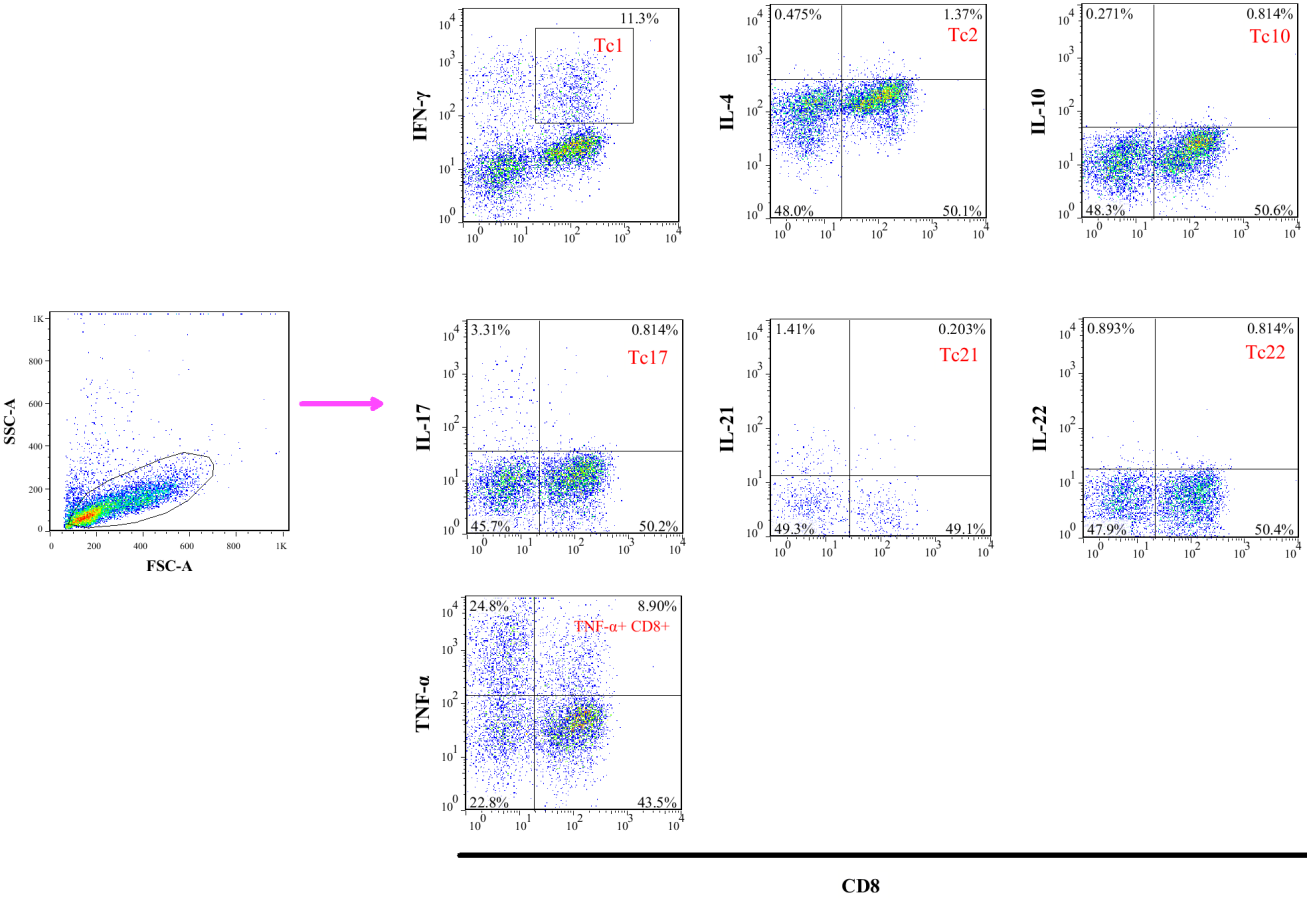
Representative gating strategy for different CD8^+^ T cell subsets. In this sample gating, cells were first gated for lymphocytes (SSC-A vs. FSC-A). The lymphocyte gate was further analyzed for IFN-γ, TNF-α, IL-4, IL-10, IL-17, IL-21 and IL-22 CD8 positive cells fractions.

The flow cytometer data analyses were conducted using the FlowJo software package v.7.6.1 (Tree Star). On average 50,000 events were acquired within the cultured PBMCs previously stimulated with PMA and Ionomycin. Lymphocytes were gated on a forward vs. side scatter dot plot and then we analyzed different CD8^+^ T cell subsets on gated cells. A representative example of gating strategy is illustrated in Fig 1. The fraction of cytokine producing CD8^+^ T cell population was assessed using one-way ANOVA in MS patients in comparison to controls. The descriptive and significances of the analysis are shown in Table 3.

**Table 3:** Descriptive statistics of different CD8^+^ T cell subsets in MS patients compared to controls.

*CD8+ T cell subsets*	*RRMS in Relapse phase*	*RRMS in Remission phase*	*SPMS*	*PPMS*	*HCs*	*P-value* ^a^
CD8^+^ IFN-γ^+^ (Tc1)	11.06 ± 6.1	13.36 ± 6.7	14.5 ± 8.6	7 ± 1.8	5 ± 5.2	0.03*
CD8^+^ IL-4^+^ (Tc2)	0.82 ± 0.4	0.86 ± 0.4	1.4 ± 1.7	0.29 ± 0.2	0.55 ± 0.2	0.22
CD8^+^ IL-10^+^ (Tc10)	0.64 ± 0.5	0.64 ± 0.5	0.36 ± 0.2	0.43 ± 0.3	0.8 ± 0.8	0.17
CD8^+^ IL-17^+^ (Tc17)	2.27 ± 2.2	1.3 ± 1.08	0.94 ± 0.7	0.54 ± 0.4	0.65 ± 0.3	0.03*
CD8^+^ IL-21^+^ (Tc21)	0.83 ± 0.6	1.17 ± 0.9	0.32 ± 0.2	0.49 ± 0.6	0.4 ± 0.5	0.05*
CD8^+^ IL-22^+^ (Tc22)	0.70 ± 0.6	0.42 ± 0.2	0.34 ± 0.2	0.33 ± 0.3	0.54 ± 0.2	0.19
CD8^+^ TNF-α^+^	15.41 ± 7.5	16.6 ± 6.5	16.1 ± 6.6	7.8 ± 2.1	5.5 ± 5.4	0.002*
CD8^+^ IFN-γ^+^ TNF-α^+^	30.6 ± 18.26	33.2 ± 17.1	29.6 ± 12.8	30.1 ± 7.6	11.8 ± 16.7	0.10
CD8^+^ IFN-γ^+^ IL-10^+^	0.76 ± 0.9	0.57 ± 0.6	0.53 ± 0.6	0.06 ± 0.6	0.42 ± 0.2	0.58
CD8^+^ IL-17^+^ TNF-α^+^	4.99 ± 3.7	3.56 ± 4.5	1.94 ± 1.3	2.09 ± 1.2	1.29 ± 2.3	0.12
CD8+ IFN-γ^+^ IL-17^+^ TNF-α^+^	11.65 ± 8.5	4.91 ± 2.7	3.37 ± 2.4	3.97 ± 0.4	2.25 ± 4.4	0.002*

Data are expressed as mean ± SD.

^a^ P values were calculated using One-Way ANOVA.

* shows significant P values.

### Enhancement of IFN-γ, TNF-α and IL-21 producing CD8 positive T cells in patients with multiple sclerosis

**Fig 2:**
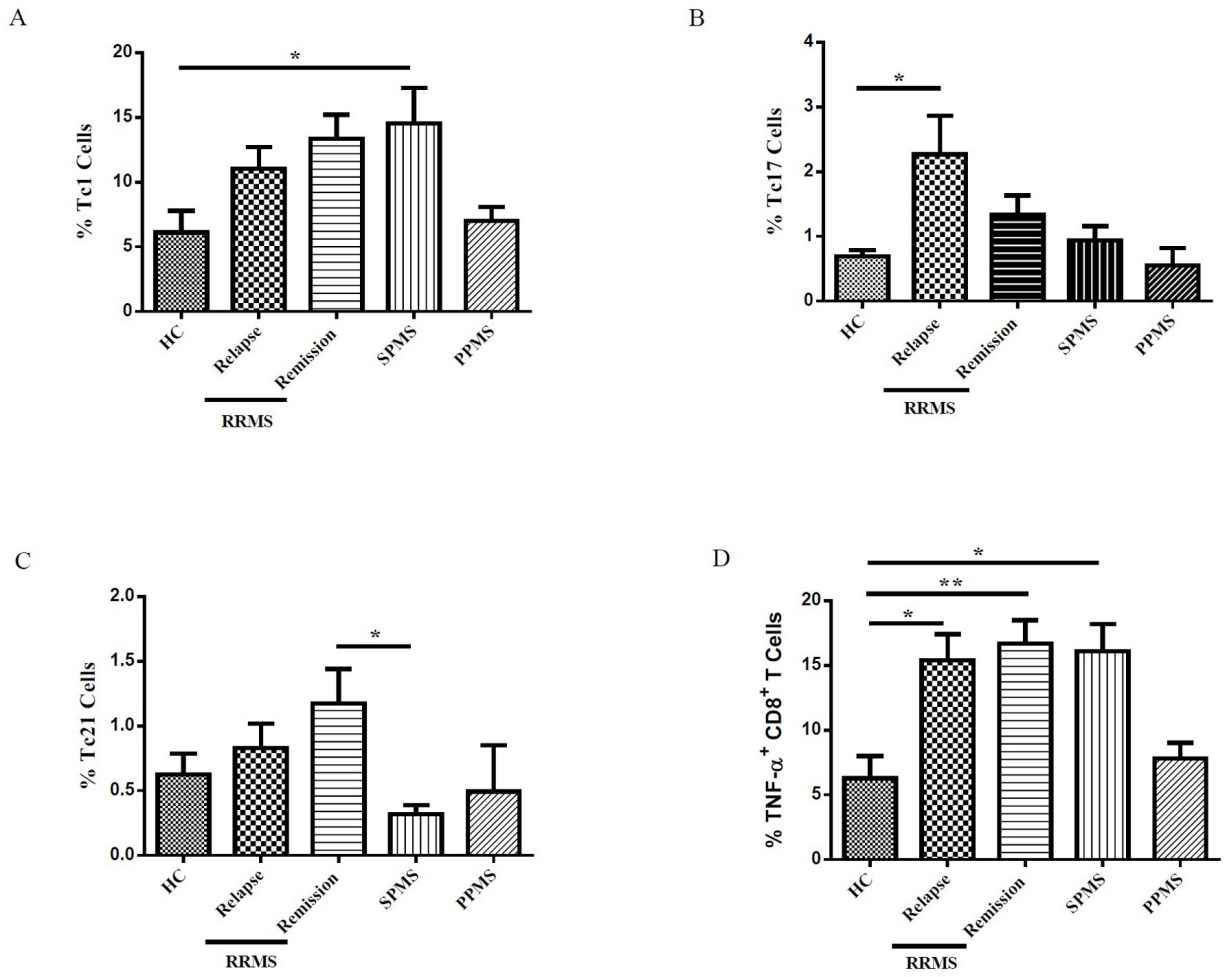
Cytokine expression by CD8^+^ T cell subsets in multiple sclerosis patients with different patterns of clinical progression. The percentage of IFN-γ (Tc1) (A), IL-17 (Tc17) (B), IL-21 (Tc21) (C) and TNF-α (D) producing CD8^+^ T cells in HCs, RRMS patients in relapse and remission phase, SPMS and PPMS patients. One-way ANOVA was used to test for differences between the groups. Tukey’s test was performed for subsequent pairwise comparison. * P-value <0.*05*, ** P<0.*01*.

First, we evaluated the frequency of Tc1, Tc2, Tc10, Tc17, Tc21, Tc22 and TNF-α producing CD8^+^ T cells in studied groups. We observed a significant increase in the percentage of Tc1 and Tc17 cells in SPMS and relapse phase of RRMS patients respectively (P = 0.04) compared to controls (Fig 2A and 2B). Interestingly, our data revealed a significant increase in the frequency of Tc21 cells in remission phase of RRMS compared to SPMS patients (P = 0.03) (Fig 2C). Furthermore, the percentage of TNF-α producing CD8+ T cells in relapse and remission phase of RRMS and SPMS patients were higher than controls (P = 0.01, P = 0.004, P = 0.01 respectively) (Fig 2D).

### The percent of IFN-γ^+^ TNF-α^+^ IL-17^+^ CD8^+^ T cells increased in relapse phase of RRMS patients

**Fig 3:**
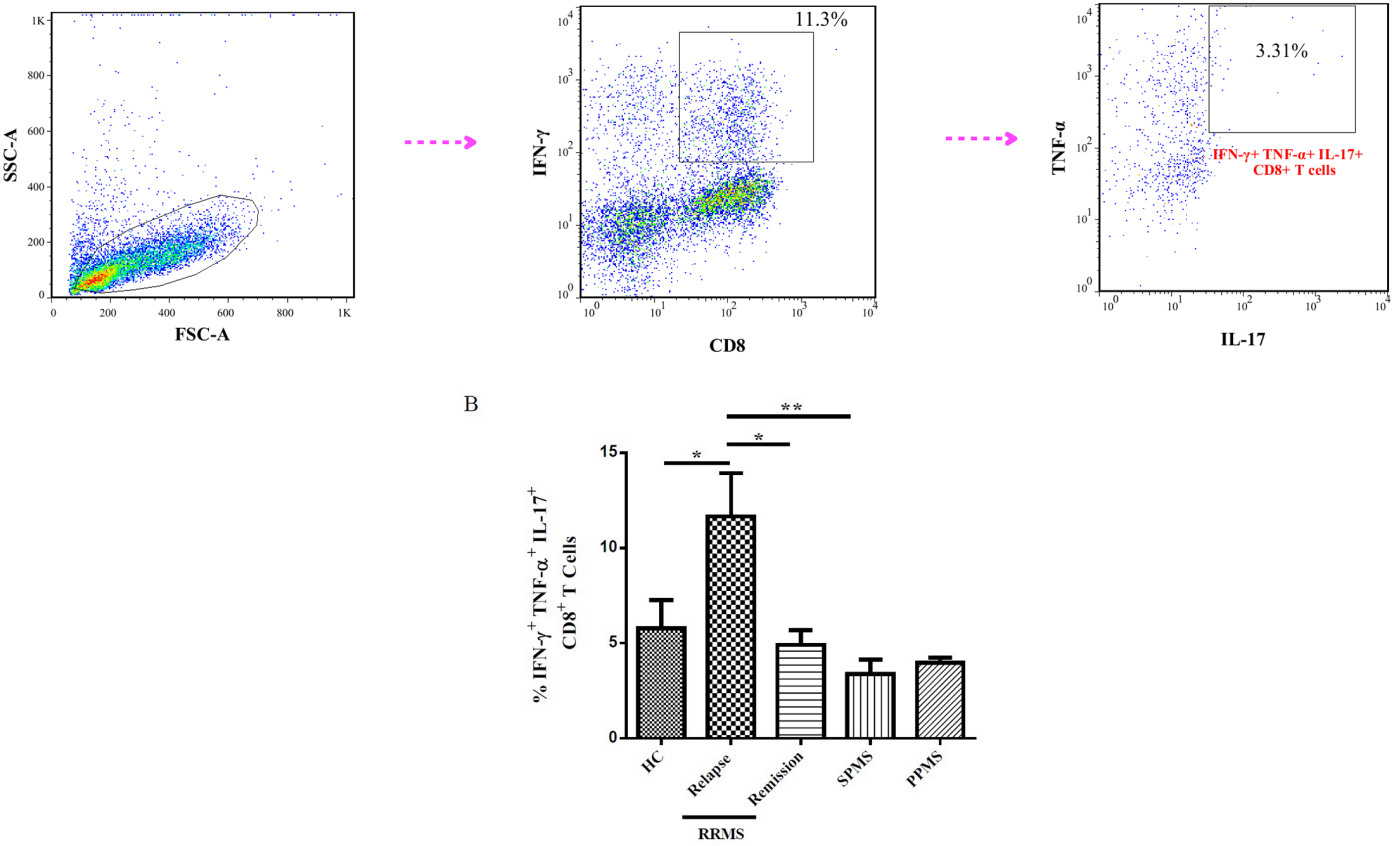
The proportion of IFN-γ^+^ TNF-α^+^ IL-17^+^ CD8^+^ T cells subset in multiple sclerosis patients with different patterns of clinical progression. (A) Flowcytometric characterization of CD8^+^ IFN-γ^+^ TNF-α^+^ IL-17^+^. (B) Proportions of CD8^+^ T cells positive for the indicated cytokines. Results are shown as bar graph. One-way ANOVA followed by a subsequent Bonferroni multiple pairwise comparison test to examine the differences between the groups. * P-value <0.*05*, ** P<0.*01*.

We next analyzed CD8^+^ IFN-γ^+^ TNF-α^+^ IL-17^+^ T cells frequency in PBMCs isolated from MS patients and HCs. We assessed TNF-α^+^ IL-17^+^ T cells on CD8^+^ IFN-γ^+^ gated cells. The gating strategy is illustrated in Fig 3A. Our findings clearly demonstrated that the percent of CD8^+^ IFN-γ^+^ TNF-α^+^ IL-17^+^ T cells increased in relapse phase of RRMS patients compared to remission phase, SPMS patients and healthy controls (P = 0.01, P = 0.004 and P = 0.02 respectively) (Fig 3B).

### Increased TGF-β serum level in secondary progressive and remission phase of RRMS patients

**Fig 4:**
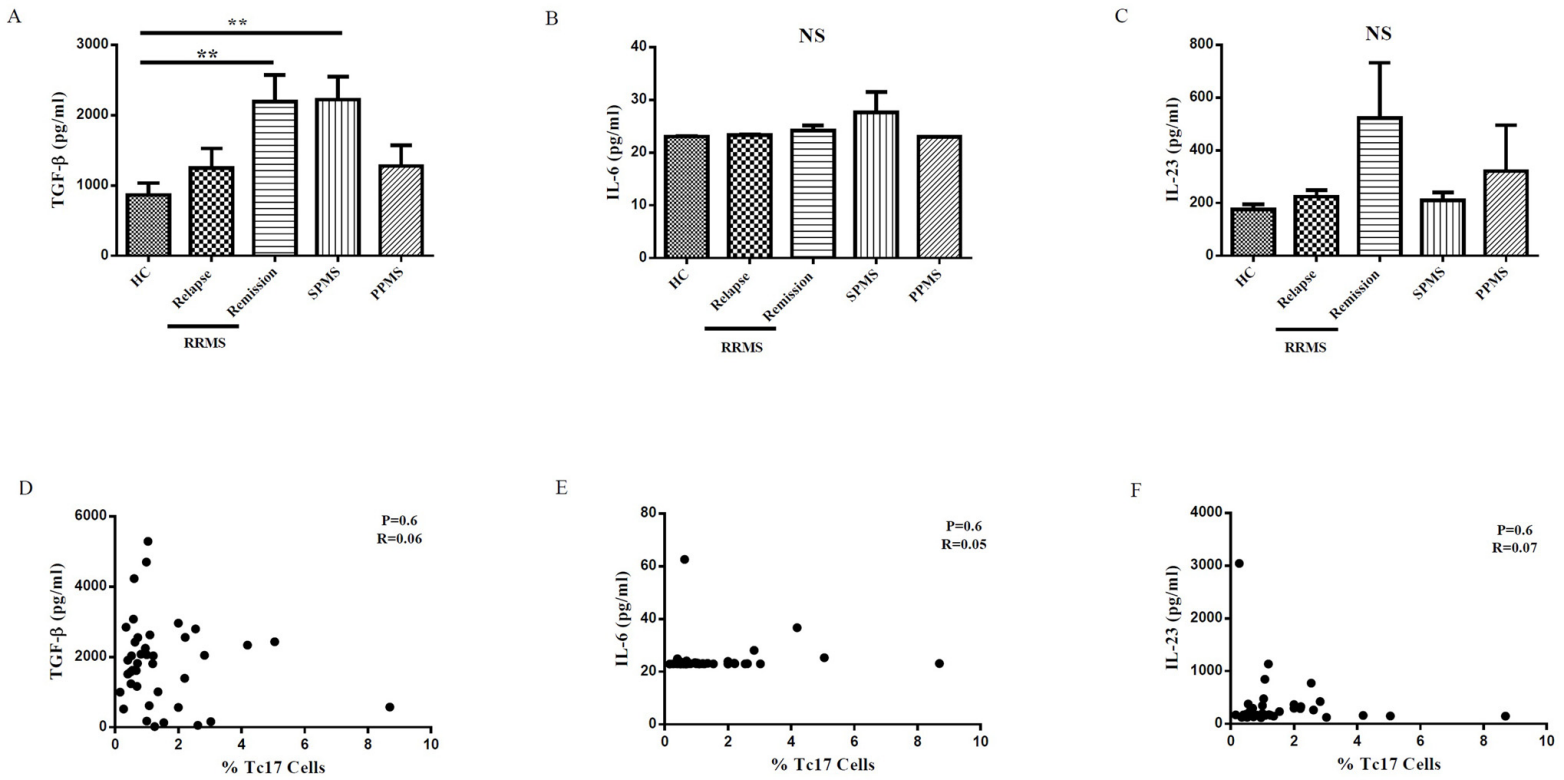
The serum cytokine levels of TGF-β, IL-6 and IL-23 was measured by ELISA in MS patients and healthy controls. Comparison of TGF-β (A), IL-6 (B), IL-23 (C) cytokines concentrations between HC, RRMS patients in relapse and remission phase, SPMS and PPMS patients. Pearson’s correlation and regression tests between TGF-β (D), IL-6 (E), IL-23 (F) and Tc17 cells in MS patients. One-way ANOVA was used to test for differences between the groups. Subsequent multiple comparison pairwise test was Bonferroni. * P-value <0.*05*.

Because of elevated frequency of Tc17 in relapse patients, we measured the relative quantity of TGF-β, IL-6 and IL-23, cytokines involved in Tc17 cells development, in sera from RRMS, SPMS and PPMS patients as well as sex and age-matched healthy control group. We observed augmented TGF-β concentration in SPMS and remission phase of RRMS patients compared with those of healthy donors (P = 0.05 and P = 0.03, respectively) (Fig 4A). The serum levels of IL-6 and IL-23 did not significantly differ between patients and controls (Fig 4B and 4C). There was no significant correlation between TGF-, IL-6 and IL-23 concentrations in serum and Tc17 cells frequency (Fig 4D, 4E and 4F).

## Discussion

CD4^+^ T helper cells have traditionally been considered as the most prominent cells in MS immunopathogenesis [26]. Although several treatment strategies have been developed in order to reduce CD4^+^ T cells, they did not show complete beneficial to alleviate the disease [27]. In contrast, depletion of both CD8^+^ and CD4^+^ T cells led to major reductions in relapses and new lesions [28]. Several studies have been performed to determine the importance of pathogenic CD8^+^ T cells in MS patients but the role of CD8^+^ T cell subsets in MS patients is less studied. In the present study, we report the frequency of different cytokine producing CD8^+^ T cell populations and serum level of Tc17 related cytokines in MS patients with various clinical patterns.

First, we showed higher percentage of IFN-γ expressing CD8^+^ T cells (Tc1) in SPMS patients in comparison to controls. However, there was no significant difference in Tc1 cells frequency between other groups. The role of Tc1 cells in the pathogenesis of MS has been extensively studied. Our data are consistent with the results reported from Purificacio´n de Castro [14] and Killestein et al. [16]. Besides these studies, Becher B, et al [18] also observed an increasing trend in the proportion of peripheral blood CD8^+^ T-cell subsets expressing IFN-γ in SPMS patients. While these data showed the significance of these CD8^+^ T subsets in SPMS, the exact role of Tc1 cells in the disease progression is still not completely understood.

CD8^+^ T cells mediate effector functions not only through the IFN-γ production, but also through TNF-α. It is well established that TNF-α is a proinflammatory cytokine. In contrary, there is substantial evidence that TNF-α regulates immunity by maintenance of central and peripheral tolerance [29]. It has been shown that CD8^+^ T cells from MS patients expressed an increased expression of IFN-γ, IL-2 and TNF-α mRNAs [30, 31]. Our results showed an increased level of TNF-α producing CD8^+^ T cells in RRMS and SPMS patients. While initial reports demonstrated abrogation of EAE via administration of TNF-α inhibitors [32], clinical trials identified MS exacerbation following soluble TNFR1-Ig treatment [33]. Recently, Kruglov et al. [34] revealed a dual role of TNF during EAE. Therefore, it may need further studies on the function of CD8^+^ TNF-α^+^ cells associated to their source in order to uncover its pathogenic and protective involvement in the MS.

Another CD8^+^ T cell subset is Tc2 cells which can be distinguished within CD8^+^ cells by producing IL-4 [35]. The role of IL-4-producing CD8^+^ T cells have been determined in several autoimmune disease. However, few studies investigated the role of Tc2 cells in MS. In this respect, we evaluated the frequency of Tc2 cells in MS patients. Our finding showed no difference of Tc2 cells percentage between subjects. Most studies regarding the Tc2 cell percentages in MS patients showed consistent findings [17, 18]. According to our knowledge there is just one study pointed to the significantly increase of CD8^+^ T cells producing IL-4 cells in PPMS patients compared to RRMS and SPMS patients [16], which do not support our results. These data may suggest that Tc2 cells may not the feature of multiple sclerosis.

We also found in this study no significant differences in Tc10 cells (CD8^+^ IL-10^+^) between studied groups. While Peelen et al. [36] showed higher levels of IL-10 in RRMS patients, others identified a reduction in Tc10 cell frequency in RRMS patients [16]. The differences between the studies may be due to the discrepancy in patients’ selection criteria.

Recently the focus has shifted toward a novel T cell subset, Tc17 cells, as the principal player of autoimmune disorders. Tzartos et al. reported for the first time equal proportion of IL-17 secreting CD4^+^ and CD8^+^ T cells in active MS lesions [19]. Our results, similar to the Wang H, et al. finding [20], illustrated the abundance of Tc17 cells in relapse phase of RRMS patients compared to HCs. Huber M, et al. showed that Th17 and Tc17 cells were not produced in IRF deficient mice, found to be resistant to EAE, and adoptive transfer of Tc17 cells could induce EAE. They also revealed that Tc17 subsets contribute to the CNS autoimmunity by supporting Th17 cell pathogenicity [37]. This enrichment of IL-17 producing CD8^+^ T cells in MS patients at relapse phase may evidence the importance of these cells in mediating MS development. Since TGF-β, IL-6 and IL-23 orchestrated initial Tc17 differentiation from naive T cells [38], we assessed the serum level of these Tc17-related cytokines in patients and healthy subjects. We demonstrated that both remission RRMS and SPMS patients have significantly increased serum TGF-β in comparison to controls. Similar to our study, there are reports showing a significantly higher cerebrospinal fluid [24] and serum TGF-β levels in MS patients in remission [23]. However, in contrast to our results, Rollnik et al. found a reduced level of TGF-β in the serum of MS patients [24]. It is well shown that on one hand, TGF-β is an immune regulatory cytokine which can help the differentiation of T regulatory cells (Tregs) [39] and as a result modify autoimmunity in MS. On the other side, it is well established that differentiation of IL-17 producing T cell required TGF-β [40] which represent the pro-inflammatory role of this cytokine in MS. The augmented level of this cytokine seen in remission phase of MS bring up this cytokine as a good option in the treatment of MS. Since we observed no correlation between TGF-β serum level and the frequency of Tc17 cells, it appears that perhaps this cytokine exert its negative affect in MS by stimulating other cells, the ones need more research. Despite the discrepancy in TGF-β level, we identified no significant difference in IL-6 and IL-23 serum concentration in MS patients and controls. Most articles regarding the level of two latter cytokines, pointed to augmentation of these cytokine in MS [41–43]. Overall, the analysis of Tc17-related cytokines in blood have resulted in controversial data which may explained by the different experimental conditions used.

Furthermore, the present study for the first time demonstrated increased percentages of IL-21^+^ CD8^+^ T cells (Tc21) in remission course of RRMS. Interleukin-21 is a member of common γ-chain family cytokine which produced by few immune cell types including Th17, T-follicular helper (TFH) and NKT cells [44]. Also it is well established that CD8^+^ T cells could be another source of IL-21 [45]. IL-21 is a pleiotropic cytokine and its biological effects depend on the type and developmental stage of the target cell [46]. Whereas IL-21 drives autoimmunity by expanding and activating pathogenic T cell subsets, NK cells and promoting antibody production by B cells [47], it can also inhibit T cell responses following decrease antigen-presenting cells (APC) function [25, 48]. Tzartos et al. also showed IL-21 and IL-21 receptor expression in lymphocytes and neurons in MS brain [49]. In the latter study IL-21 is restricted to CD4^+^ Cells. These finding may implicate a role for Tc21 in MS pathogenesis which warrants further exploration. In another words, understanding the exact function of IL-21 produced by CD8^+^ T cells in neuro-inflammatory disease such as MS needs to identify the exact target of this cytokine.

Increased concentration of Interleukin-22 (IL-22) have been recently viewed in MS patients [50]. IL-22 is a member of IL-10 cytokine family that was discovered to be mainly produced by CD4^+^ T cells and associated with several autoimmune diseases such as inflammatory bowel diseases (IBD), psoriasis and systemic lupus erythematosus (SLE) [51, 52]. Moreover, IL-22, along with IL-17, involved in pathology of MS by affecting the blood brain barrier (BBB) integrity [53]. Similarly to CD4^+^ T cell compartments, IL-22 producing CD8^+^ T cells are now recognized [50]. Although we addressed higher proportion of Tc22 cells in RRMS patients in active phase, it was not significant. It seems that future studies with larger sample size needed to exactly clarify the role of these CD8^+^ T cell subsets.

Finally, we addressed IFN-γ^+^ TNF-α^+^ IL-17^+^ CD8^+^ T cells as triple producer cytotoxic T cells in PBMCs. Our study described for the first time significantly higher frequency of triple producer Tc cells in RRMS patients in relapse stage comparing to healthy controls, SPMS and remission phase of RRMS patients. Several reports have clearly demonstrated that Tc17 cells are indeed plastically converted into IFN-γ-producing Tc1-like cells that act as pathogenic effector cells [54]. However, the mechanisms that allow CD8^+^ T cells to simultaneously produce IL-17 and IFN-γ not revealed. Recently Tajima M, et al. proposed that Tc17 cells in presence of IL-12 converted into cytotoxic Tc17/IFN-γ^+^ cells [55]. In addition, Prat A, et al. noted a subpopulation of human IL-17^+^ CD4^+^ T lymphocytes producing IFN-γ in blood of MS patients crossed the BBB more efficiently [56]. It appears that targeting IFN-γ^+^ TNF-α^+^ IL-17^+^ CD8^+^ T cells may provide new therapeutic approach for MS.

## Conclusion

In this paper we provide some new insights in the frequency of different CD8^+^ T cell subsets in MS patients with different clinical pattern. To our knowledge, this is the first paper demonstrated increased frequency of IL-21 producing CD8^+^ T cells in remission phase of RRMS. In addition, our results show higher frequency of both Tc17 cells and triple producer CD8^+^ T cells (IFN-γ^+^ TNF-α^+^ IL-17^+^ CD8^+^ T cells) cells, for the first time, in relapse phase of RRMS which amplify the idea that Il-17 as one of the key player in MS pathogenesis. However, more studies are required to clarify the exact function of these CD8^+^ T cell subsets in the disease.
